# Mapping the local particle plasmon sensitivity with a scanning probe

**DOI:** 10.1039/c6nr05800k

**Published:** 2016-08-30

**Authors:** Markus K. Krug, Gernot Schaffernak, Martin Belitsch, Marija Gašparić, Verena Leitgeb, Andreas Trügler, Ulrich Hohenester, Joachim R. Krenn, Andreas Hohenau

**Affiliations:** a Institute of Physics , University of Graz , 8010 Graz , Austria . Email: markus.krug@uni-graz.at

## Abstract

We probe the local sensitivity of an optically excited plasmonic nanoparticle by changing the local dielectric environment through a scanning glass fiber tip. Recording the particle plasmon scattering spectrum for each tip position allows us to observe spectral resonance shifts concurrent with changes in scattering intensity and plasmon damping. For the tip-induced spectral shifts we find the strongest sensitivity at the particle edges, in accordance with the spatial plasmonic field profile. In contrast, the strongest sensitivity occurs at the center of the particle if the scattering intensity is probed at the short wavelength slope of the plasmon resonance instead of the resonance position. This bears important implications for plasmonic sensing, in particular when done at a single light wavelength.

## Abstract 2

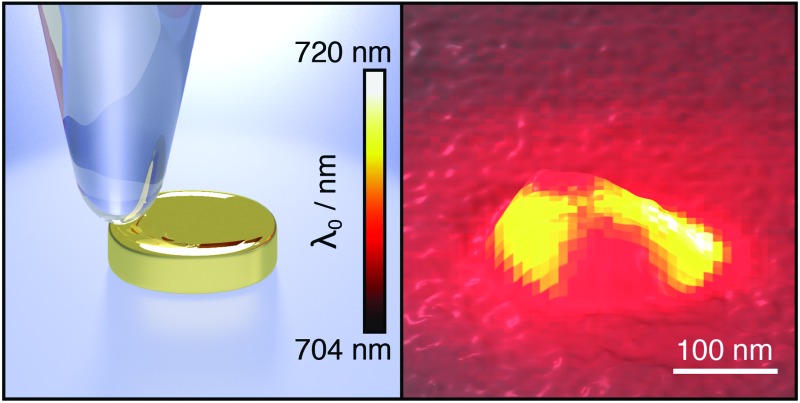
A gold nanoparticle is scanned with a dielectric tip while optical scattering spectra are acquired for each tip position to map plasmon resonance changes.

## 1. Introduction

Metal nanoparticles sustain localized surface plasmon resonances (LSPRs) in the visible or near-infrared spectral range. Their spectral positions are set by the optical constants of the metal, the particle geometry, and the dielectric environment.^1,2^ LSPR fields are highly confined to the particle surface, decaying over a distance that corresponds to a fraction of the free-space wavelength. It is thus only a nanoscale volume in the immediate particle environment that contributes to the LSPR properties. This effect is used for the refractometric (label-free) detection of analytes down to the single molecule level, usually probed in terms of a spectral redshift due to analyte binding.^3–8^

The wavelength shift induced by an analyte molecule depends on the local electric field intensity of the LSPR, which, in general, is nonuniform around the particle.^9^ Therefore, the resulting wavelength shift is nonuniform, in regions of particularly high local electric field intensity (“hot spots”) the sensitivity is greatly enhanced. This point becomes especially important when the number of analyte molecules (or diffusion to the detection area) is severely limited. This case demands the maximum signal from an individual analyte molecule, which should therefore be captured in a plasmonic hot spot to generate the largest possible signal.^10^ In this way, individual binding events can be observed,^7^ thus enabling the study of single molecular interactions, provided that the sensitivity profile of a given nanoparticle is known beforehand. In addition, other LSPR applications as, *e.g.*, coupling to quantum emitters also require an understanding of the spectral LSPR response to local refractometric changes. The search for positions on a nanoparticle's surface with maximum sensitivity to local dielectric changes has attracted much interest.^9,11–14^ Experimentally, this was accomplished by electron-beam lithographic^15,16^ or scanning probe based^17^ positioning of dielectric nanostructures at defined positions on or around a plasmonic nanoparticle and tracking the resulting spectral LSPR shifts. Electron microscopy based spectroscopy techniques (electron energy loss spectroscopy,^18^ cathodoluminescence^19^) provide superior resolution of the plasmonic mode density, however they cannot account for analyte-induced LSPR shifts. In this work, we implement a setup for the three-dimensional mapping of a plasmonic nanoparticle's LSPR response to a local dielectric nanostructure by means of a nanometric glass-tip which is position-controlled in the nanoparticle's proximity. Our approach goes back to the work of Kalkbrenner *et al.*,^20^ who used a similar setup to demonstrate that a dielectric tip only weakly influences the plasmonic modes of a gold nanosphere. It is however exactly this “weak” influence that we investigate here, to show that the near field coupling of tip and plasmonic nanoparticle leads to tip-position dependent modifications of LSPR wavelength, -scattering intensity and -peak-width, which spatially vary on the scale of the tip radius. If for each tip position a scattered light spectrum is acquired, local response maps based on different spectral parameters can be created. Depending on the plotted quantity, these response maps show fundamental differences.

## 2. Experimental

**Fig. 1:**
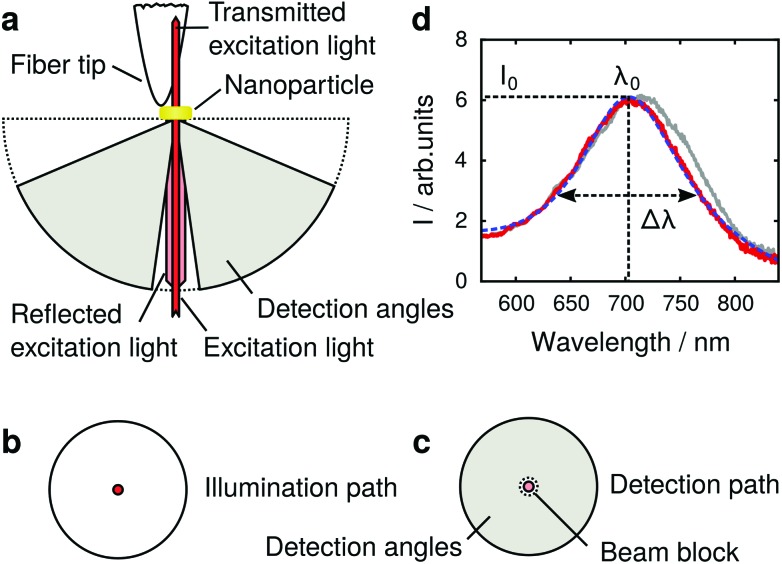
Dark field single plasmonic particle spectroscopy. (a) Schematic of the setup and detection and excitation angles, (b) and (c) depict conjugates of the back focal plane of the microscope objective in the illumination and detection light paths. (d) Experimental scattering spectra of a gold nanoparticle with a dielectric tip positioned far away (solid red) and close (solid gray) to the nanoparticle. The data is fitted with eqn (1) (dashed blue).

In our experiment we measure optical scattering spectra of single plasmonic nanoparticles with a dark field setup, using an oil immersion objective (Nikon, 60×, numerical aperture 1.4) for illumination and detection from the substrate side (Fig. 1a). A white-light supercontinuum laser (Fianium, WL-SC-400-4) is used for illumination, with infrared radiation removed by a band-pass filter (500–1000 nm). The laser beam is linearly polarized and spatially filtered through a pinhole. The position of the pinhole coincides with the conjugate back focal plane (Fig. 1b) of the microscope objective, creating a normal incidence wide field illumination of the sample.

**Fig. 2:**
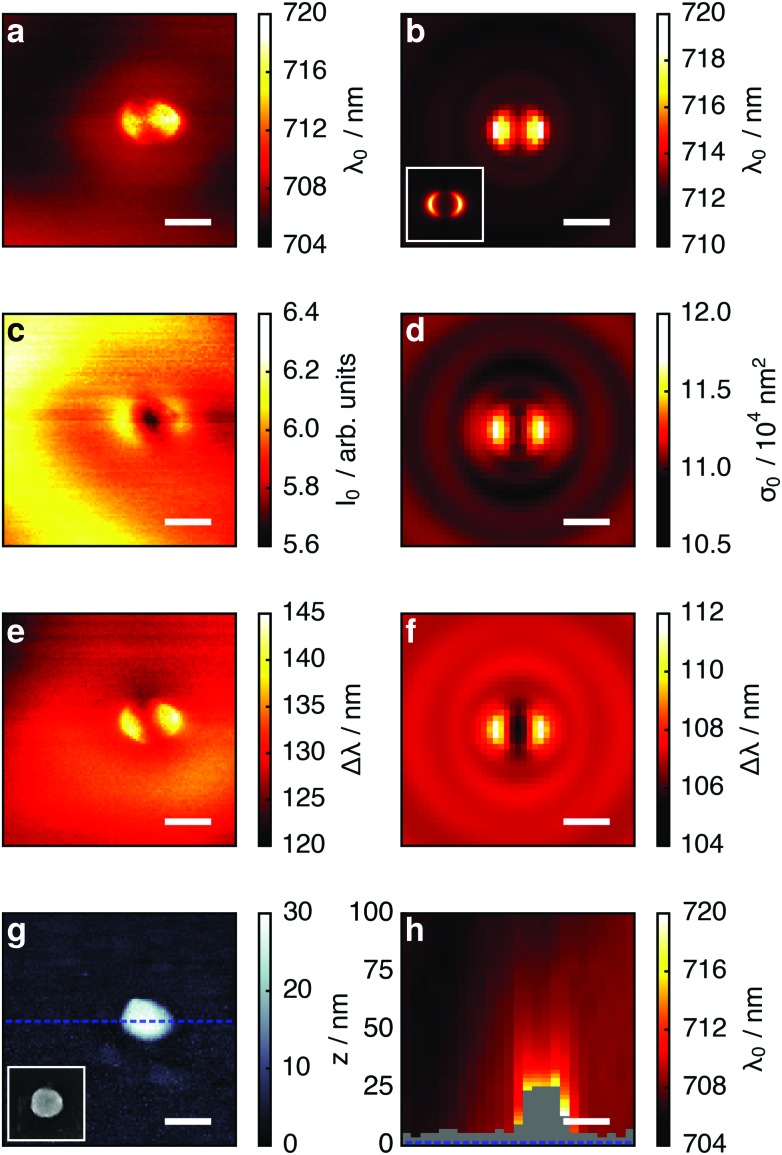
Maps of tip-induced LSPR changes of a gold nanoparticle; left column experiment, right column simulation: (a, b) LSPR wavelength; (c, d) maximum scattering intensity/scattering cross-section; (e, f) LSPR peak width. The inset in (b) depicts the electric near-field intensity of an unperturbed nanoparticle in a plane 4 nm from its surface (same scale as main figure). Panel (g) depicts the simultaneously acquired nanoparticle topography. The inset depicts a scanning electron microscope image of an identically fabricated nanoparticle after coating with 3 nm Cr for electric conductivity. Panel (h) shows the LSPR wavelength in a vertical plane through the dotted blue line in (g). The polarization direction is horizontal throughout. The scale bar is 200 nm, the height in (h) is directly indicated on the left side of the panel.

Light scattered by the plasmonic nanoparticle and reflected by the glass/air interface of the substrate is collected by the microscope objective. The specularly reflected excitation beam is removed by a small beam block (numerical aperture 0.2) in the center of the objective's conjugate back focal plane in the detection path (Fig. 1c) after the illumination beam-splitter. Thus, a dark field illumination scheme is realized, which as well rejects light reflected from the scanning probe and its holder. The scattered light passes the beam block and is imaged onto the slit of an imaging monochromator (Andor SR-303i) and is detected by an EMCCD camera (Andor IXon DV885LC). The integration time for one scattering spectrum is 10 ms, and one single measurement typically consists of 100 × 100 spectra (measurement points). The recorded spectra are background corrected, and the direct reflection at the glass/air interface is measured without beam block to serve as the reference. As only the lower half space, *i.e.*, the glass side of the sample, is used for optical illumination and detection, the upper (air facing) half space remains free for the scanning probe. We use etched glass fiber probes^21^ (tip radius 40 ± 5 nm, as determined from the broadened topography in comparison to the electron microscope image in Fig. 2g) mounted on a homemade tuning fork system,^22^ controlling the tip–sample distance with shear force feedback.

It should be noted that our setup is akin to a scattering-type scanning near field optical microscope, where a sharp tip scatters optical near fields into the far field for detection.^23^ Such microscopes have been used to, *e.g.*, probe plasmonic near fields with dielectric tips under the premise that the influence of the tip is negligible.^24–26^ In contrast, our system relies on the modification of the plasmonic system by the dielectric tip, thus probing the combined system of tip and plasmonic particle for each tip position.

To extract the resonance frequency *ω*_0_, the resonant scattering intensity *I*_0_, and the resonance width Δ*ω* from the scattering spectra, and to analyze their tip-induced changes, the spectra are fitted by a modified Lorentzian line shape, adapted from ref 27:1
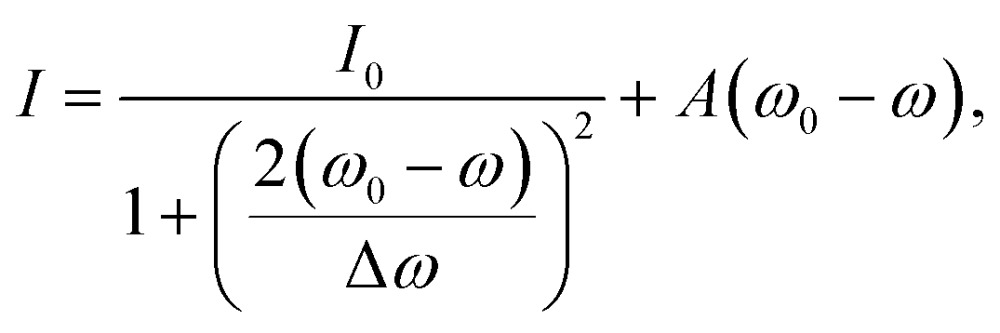
with *I* intensity, *ω* frequency; Δ*ω* is defined as the full-width at half-maximum (FWHM). The linear term *A*(*ω*_0_ – *ω*) considers in a lowest-order approximation the contribution of the frequency dependent dielectric function of the gold, which is responsible for the asymmetric peak shape with more intensity at the short wavelength side (Fig. 1d). For clarity, resonance position and width are converted to a wavelength scale (*ω*_0_ → *λ*_0_, Δ*ω* → Δ*λ*). The fit parameters *λ*_0_, *I*_0_ and Δ*λ* are then arranged in two-dimensional maps according to the tip position on the sample (Fig. 2). Comparing the spectral maps with the topographical map (Fig. 2g) that is simultaneously acquired, the spectral changes are matched to the relative position of tip and nanoparticle. As sample we use individual gold nanoparticles fabricated by electron beam lithography on glass substrates.^28^ The quasi-identical nanoparticles have an elliptical footprint with lateral dimensions of 140 ± 5 nm × 120 ± 5 nm and a height of 25 ± 3 nm. When excited along the long particle axis the LSPR peak is at 705 nm (Fig. 1d).

## 3. Simulation

We corroborate our experimental results with numerical simulations for the full Maxwell's equations performed with the MNPBEM toolbox,^29^ including substrate effects^30^ and modeling the particle as elliptical cylinder (with rounded top edge, see Fig. 4) according to the experimental dimensions. For the gold dielectric function we use tabulated values obtained from optical experiments,^31^ and the total number of boundary elements was 1380. The discretization grid for the Green's function table, important for convergence, was set *n*_z_ = 60 and *n*_r_ = 120 for vertical and radial directions, respectively.^30^ Scattering cross sections *σ* were calculated for a plane wave excitation from the substrate side at normal incidence (25 discrete wavelengths, 40 × 40 tip positions). The glass tip was approximated as a dielectric sphere (*ε* = 2.25) with a radius of 40 nm, assuming that due to the rapid plasmonic field decay only the very end of the tip is of importance. This assumption was verified by simulations for an extended conical tip, that showed only slightly different resonance shifts but no qualitative differences. In the simulations the dielectric sphere was kept at a constant distance of 3 nm to the nanoparticle/glass surface which is the typical experimental tip height as determined from tip-to-sample approach curves. Unless otherwise specified, standard parameters of the MNPBEM14 toolbox are used.^30^

## 4. Results and discussion

Fig. 2 depicts the tip-induced LSPR changes as maps of resonance wavelength, maximum scattering intensity and peak width. Starting with the resonance wavelength (Fig. 2a), we find a detailed pattern around the nanoparticle (topography shown in Fig. 2g), confirming the local character of our tip-based measurement. The strongest resonance redshifts of approximately 14 nm are found for tip positions near the particle edges in the horizontal direction, which corresponds to the polarization direction of the exciting light. The agreement of the experimental data with the simulated map in Fig. 2b is very good. In addition, the patterns coincide well with the simulated electric field intensity for the dipolar plasmon mode of the gold nanoparticle (inset Fig. 2b). This is in accordance with the description of plasmon resonance shifts for small nanoparticles as being proportional to the local electric field intensity at the location of the dielectric perturbation.^9^

Owing to their localized nature, near-field effects are very sensitive to the details of the local dielectric environment. We attribute the slight asymmetry of the experimental maps in Fig. 2 to small asymmetries in the tip geometry, as in further experiments we found it to vary slightly from tip to tip but not when different particles were scanned with the identical tip. Uncertainties in the particle dimensions and dielectric function^31^ are likely to account for the offset between experimental (*λ* = 705 nm) and simulated resonance wavelength (*λ* = 710 nm) in absence of the tip.

**Fig. 3:**
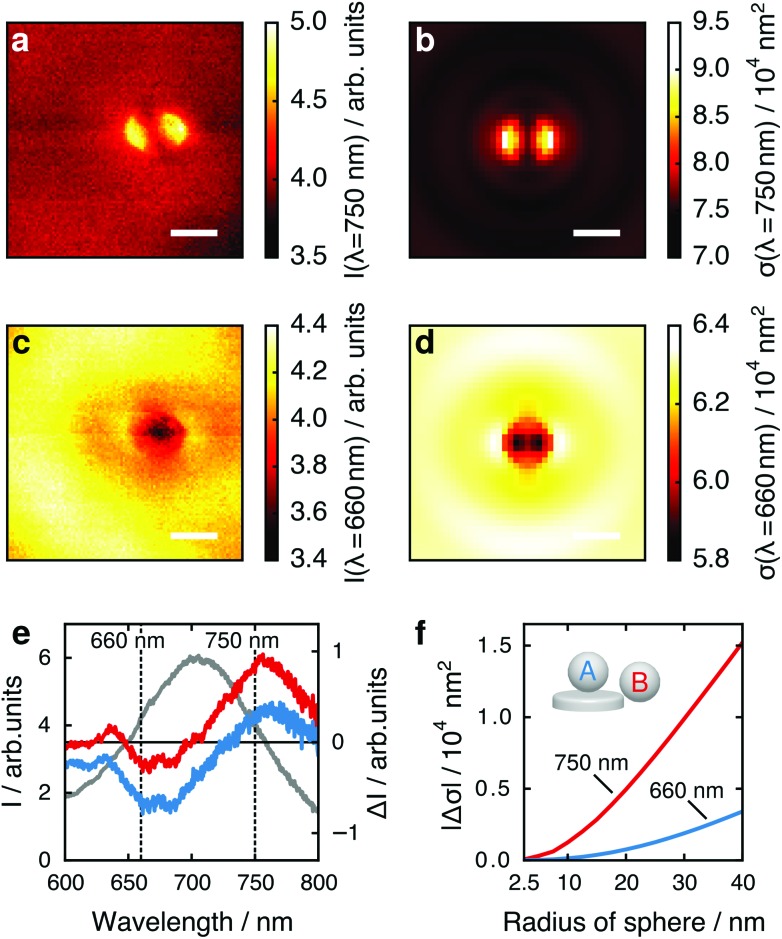
Maps of tip position dependent scattering intensity at a light wavelength of (a) 750 nm and (c) 660 nm. Corresponding simulation results are shown in (b) and (d). The scale bar is 200 nm, the polarization direction is horizontal. (e) Nanoparticle spectrum in absence of tip (gray, left *y*-axis), and spectral changes Δ*I* for tip in the center (blue) and on the edge (red). (f) Absolute values of spectral changes |Δ*σ*| simulated for several tip radii for two particular cases: the edge position yielding the highest spectral change at 750 nm (pos. B, red line), while the center position is best detected at 660 nm (pos. A, blue line).

Considering these results, we next critically examine the usual plasmonic biosensing scheme that relies on measuring extinction or scattering at a single light wavelength, which is usually chosen to lie within the steepest slope on either side of the LSPR peak (rather than considering the full LSPR spectrum, as here). A change in LSPR wavelength due to a binding analyte then translates to a change in intensity of the transmitted or scattered light at the probe wavelength. In Fig. 3 we plot the maps of the tip position dependent scattering intensity for fixed wavelengths of 750 nm (long wavelength slope of the LSPR peak around 705 nm) and 660 nm (short wavelength slope). At 750 nm (Fig. 3a and b) the strongest change in scattering intensity appears with the tip positioned at the left or right particle edge. This map thus resembles the map of tip-induced LSPR shifts (Fig. 2a and b), as expected: an LSPR redshift leads to stronger scattering for a wavelength at the long wavelength slope of the LSPR maximum. Conversely, for a fixed wavelength at the short wavelength slope of the LSPR peak we thus would expect an inverted map: an LSPR redshift leads to reduced scattering at this wavelength, and regions of large LSPR shift should therefore correspond to regions of low scattering intensity. Remarkably, the maps for a wavelength of 660 nm (Fig. 3c and d) show a completely different pattern, with the lowest scattering intensity appearing when the tip is centered above the particle.

To compare the maximum local response to the presence of the tip at the two wavelengths, we consider the change in scattered intensity when the tip is moved from a position far away from the nanoparticle to the positions of maximum tip influence (over the particle edges in Fig. 3a and b or over the particle center in Fig. 3c and d). In Fig. 3e we plot the change in intensity Δ*I* when the tip is brought close to the nanoparticle as a measure for sensing performance.^27^ The best contrast for the center of the gold nanoparticle (blue) is achieved at approximately 660 nm, for the edge (red) at approximately 750 nm, with similar intensity changes. This effect is strongly size dependent. In Fig. 3f, we calculate both cases for spheres of different radii, plotting the absolute values of their calculated scattering cross section changes |Δ*σ*|. The ratio of Δ*σ* at the edge to Δ*σ* at the center increases from 4.5 for a sphere of 40 nm radius to 9.4 for a sphere of 5 nm radius, suggesting that small analytes are best detected at the nanoparticle edge. However for large analytes, *e.g.*, viruses, analyte binding sites in the particle center can cause a response quantitatively similar to binding sites on the particle edge when considering single wavelength detection schemes. This is in contrast to schemes where the response is monitored by resonance shifts only, which are considerably less sensitive to binding sites in the particle center.^32^

To develop a physical understanding of these observations, we first look at the tip-induced changes of the resonant scattering intensity *I*_0_ and -cross-section *σ*_0_ (Fig. 2c and d) and LSPR spectral width Δ*λ* (Fig. 2e and f). If the tip is positioned at the particle edge, maxima in the LSPR, the scattering intensity, and the peak width are observed. When probing at a wavelength of 750 nm all these effects add up to give a higher scattering intensity. However, at 660 nm they partly compensate each other: the redshift of the LSPR leads to a decrease whereas the larger peak width and scattering cross-section lead to an increase of the scattering intensity.

**Fig. 4:**
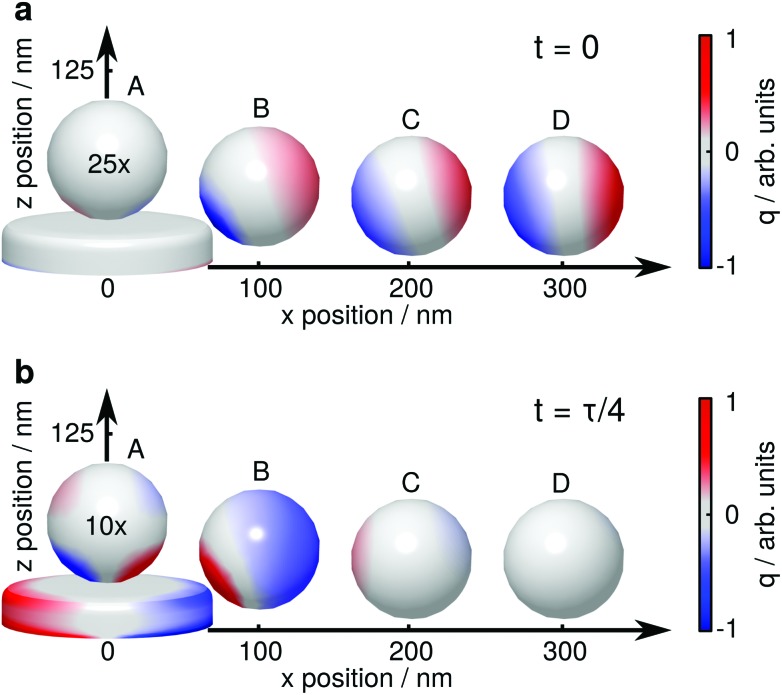
Surface charge density *q* of a gold nanoparticle and a dielectric sphere for several relative positions, (a) for *t* = 0, where the electric field of the exciting light wave has a maximum at *z* = 0, and (b) *t* = *τ*/4, where the resonantly driven polarization of the nanoparticle has a maximum. The surface charge densities are drawn at 723.1 nm, 728.1 nm, 721.5 nm and 721.5 nm (A–D), *i.e.*, where the near field of the plasmon resonance is maximum.^33^ The surface charge densities on the sphere are exaggerated 25 times (a) and 10 times (b), respectively. The influence of the sphere on the nanoparticle's surface charge distribution is barely visible in these plots, the nanoparticle is thus plotted only once.

For a more detailed explanation of the involved processes, the coupling between tip (sphere) and nanoparticle has to be considered. We visualize this coupling by plotting the surface charge densities of sphere and nanoparticle in Fig. 4. For larger distances, positions C and D, the coupling between nanoparticle and sphere is weak, and their polarizations are independently driven by the external electric field. The non-resonant polarization of the sphere is in phase with the excitation, and is maximum at *t* = 0 (panel a) and 0 at *t* = *τ*/4 (*τ* = 2π*ω* ^–1^, panel b). On the contrary, the resonant nanoparticle's polarization is π/2 out of phase and thus has a maximum at *t* = *τ*/4 and is 0 a *t* = 0.

When the sphere approaches the nanoparticle, positions A and B, the polarization of both objects is altered by their near field interaction. As for the sphere, its polarization is driven by the nanoparticle near fields (almost in phase with them), and we observe a reduction of the non-resonant polarization due to shadowing (Fig. 4a). The back-action of the sphere's polarization on the nanoparticle leads to the observed resonance shifts. The modification of the scattering intensities can be interpreted as follows: for position B the dipole moments of the sphere and the nanoparticle approximately point into the same direction, sum up constructively and thus give rise to an increased scattering (Fig. 2a and b, bright lobes at the particle edges). In contrast, for position A, the dipole moments point into almost opposite directions, sum up destructively, and thus decrease the scattering (Fig. 2c and d, dark spot at the particle center).

We now turn to the tip-induced changes of the plasmonic peak width. This parameter is a measure for the LSPR damping, which can be separated in Ohmic and radiation damping. For a nanoparticle in the size range considered here, both are relevant. From the full simulations we can separate both processes and find that radiation damping contributes approximately 75%. In a quasi-static model, radiation damping is proportional to the scattering cross-section and Ohmic damping is proportional to its square root (*i.e.*, proportional to the induced dipole moment). The peak width map could thus be expected to be similar to that of the scattering cross-section. However, the presence of the tip also slightly modifies the field distribution within the gold nanoparticle, which causes more absorption. This is responsible for the qualitative differences between Fig. 2c–f.

Finally, we show that LSPR mapping is not limited to the sample surface, but by lifting the tip from the substrate it can be readily extended to a three-dimensional measurement. Fig. 2h shows the LSPR wavelength for tip positions in a vertical plane (through the dotted blue line in Fig. 2g). The maximum resonance shift is found close to the nanoparticle surface and decays within a fraction of the free space wavelength. We note that in this measurement the spectra were acquired at different heights above the substrate and particle so that for plotting the map we had to slightly interpolate between the data points in the vertical direction.

## 5. Conclusion

In summary, we apply a scanned glass tip to probe the spectroscopic response of a plasmonic nanoparticle to a local dielectric change of its environment. For each tip position, we record dark field scattering spectra from the combined tip/particle system. We demonstrate that the tip-induced effects on the plasmon resonance wavelength, scattering cross-section and resonance width can be separated and thus mapped individually. The largest LSPR shifts are found at the particle edges, according to the plasmonic dipole pattern.

By analyzing the changes in scattering cross-section at a fixed wavelength at the slope on either side of the LSPR peak, we find that a sensitivity map similar to that of LSPR shift is found only if the sensing wavelength is at the long wavelength slope. At the short wavelength slope, the maximum change in signal is found if the dielectric disturbance is at the center of the particle. This on first sight surprising result is explained by the interplay of resonance shift, scattering cross-section and LSPR width. As a scanning probe technique the obtained spatial resolution is governed by the shape of the tip. We suggest that commercially available, sharp (*r* < 10 nm) scanning probe tips could improve spatial resolution and allow quantitative statements. Our results demonstrate the versatility of the used approach for the detailed analysis of the impact of dielectric nanostructures (analytes) upon the properties of a plasmonic nanoparticle.
